# Impact of Nanoencapsulated Rosemary Essential Oil as a Novel Feed Additive on Growth Performance, Nutrient Utilization, Carcass Traits, Meat Quality and Gene Expression of Broiler Chicken

**DOI:** 10.3390/foods13101515

**Published:** 2024-05-13

**Authors:** Sheikh Adil, Mohammad T. Banday, Syed A. Hussain, Manzoor A. Wani, Ebtesam Al-Olayan, Amlan K. Patra, Shahid Rasool, Adil Gani, Islam U. Sheikh, Azmat A. Khan, Showkeen Muzamil

**Affiliations:** 1 Division of Livestock Production and Management, Faculty of Veterinary Sciences & Animal Husbandry, Sher-e-Kashmir University of Agricultural Science and Technology (Kashmir), Jammu & Kashmir, Shuhama 190006, Indiamanzoorwani5193@gmail.com (M.A.W.); sheikhiu@gmail.com (I.U.S.);; 2 Division of Livestock Products Technology, Faculty of Veterinary Sciences & Animal Husbandry, Sher-e-Kashmir University of Agricultural Science and Technology (Kashmir), Jammu & Kashmir, Shuhama 190006, India; dr.arshidsyed@gmail.com; 3 Department of Zoology, College of Science, King Saud University, Riyadh 11451, Saudi Arabia; 4 American Institute for Goat Research, School of Agriculture and Applied Sciences, Langston University, Langston, OK 73050, USA; 5 Council of Scientific and Industrial Research, Field Station, Bonera, Pulwama 192301, India; 6 Department of Food Technology, University of Kashmir, Jammu & Kashmir, Hazratba 190006, India; 7 Division of Veterinary Biochemistry, Faculty of Veterinary Sciences & Animal Husbandry, Sher-e-Kashmir University of Agricultural Science and Technology (Kashmir), Jammu & Kashmir, Shuhama 190006, India; showkeen.muzamil82@gmail.com

**Keywords:** broiler chicken, *Rosmarinus officinalis* L., growth performance, gene expression, meat quality, nanoencapsulation

## Abstract

This study evaluated the effect of free and nanoencapsulated rosemary essential oil (REO) as an antibiotic alternative in broiler diets on growth performance, nutrient digestibility, carcass traits, meat quality and gene expression. Four hundred twenty day-old commercial broiler chicks (VENCOBB) were randomly allocated to seven dietary treatments, each having four replicates of fifteen chicks. The dietary treatments comprised control (CON) fed a basal diet only, AB (basal diet + 10 mg enramycin/kg), CS (basal diet + 150 mg chitosan nanoparticles/kg), REO_F100_ and REO_F200_ (basal diet + 100 mg and 200 mg free REO/kg, respectively), and REO_N100_ and REO_N200_ (basal diet + 100 mg and 200 mg nanoencapsulated REO/kg, respectively). Overall (7–42 d), REO_N200_ showed the highest (*p* < 0.001) body weight gain (1899 g/bird) and CON had the lowest gain (1742 g/bird), while the CS, REO_F100_ and REO_F200_ groups had a similar gain, but lower than that of the AB and REO_N100_ groups. Feed intake was not affected by dietary treatments. Overall, the feed efficiency increased (*p* = 0.001) by 8.47% in the REO_N200_ group and 6.21% in the AB and REO_N100_ groups compared with the CON. Supplementation of REO improved (*p* < 0.05) dry matter and crude protein digestibility, with the highest values in REO_N100_ and REO_N200_. Ether extract, crude fiber, calcium and phosphorus digestibility values showed no difference among the groups. The dressing, breast, thigh % increased (*p* < 0.05) and abdominal fat % decreased (*p* < 0.001) more in the REO_N200_ group than with other treatments and CON. In breast meat quality, water holding capacity and extract reserve volume increased (*p* < 0.05) while drip loss and cholesterol content decreased (*p* < 0.05) in REO_N100_ and REO_N200_. No change was observed in the breast meat color among dietary treatments and CON. The REO_N100_ and REO_N200_ groups had reduced (*p* < 0.05) meat lipid peroxidation as depicted by the decreased levels of TBARS, free fatty acids and peroxide value compared to other treatments and CON. The expression of the Mucin 2, PepT1 and IL-10 genes was upregulated (*p* < 0.001) and TNF-α downregulated (*p* < 0.001) by dietary addition of REO particularly in the nanoencapsulated form compared with the CON. In conclusion, nanoencapsulated REO, especially at 200 mg/kg diet, showed promising results as an antibiotic alternative in improving the performance, nutrient digestibility, carcass traits, meat quality and upregulation of growth and anti-inflammatory genes.

## 1. Introduction

Over the years, antibiotic growth promoters (AGPs) at sub-therapeutic dosage have successfully been incorporated in the diet for promoting growth and protecting health by way of controlling gastrointestinal infections and modification of intestinal microbiota of poultry [1,2,3]. However, their indiscriminate use has resulted in the emergence of antibiotic resistance in addition to residues of these drugs in animal products [4,5]. In view of this, the European Union (EU) banned the use of antibiotics to be used as growth promoters in food animals [6]. The U.S. Food and Drug Administration (FDA) issued guidelines on prohibiting labelling of drugs for growth promotion in food animals [7]. This subsequently increased consumer awareness, which led researchers to explore other safe alternative feed additives for poultry [8]. Among various alternatives, phytogenic feed additives gained popularity among the research community because these are generally recognized as safe (GRAS) [9].

Phytogenic feed additives (PFAs) include a range of spices, herbs, and their extracts, which contain a plethora of bioactive compounds including flavonoids, phenolic compounds, and essential oils (EOs). PFAs have positively affected the performance and health of poultry by exerting antibacterial, immune-oxidant properties and gut manipulation [10]. EOs are lipophilic, volatile compounds extracted from plants, and exert various biochemical, physiological, pharmacological, and antimicrobial actions in the body [11,12]. EOS have been reported to have performance-enhancing effects in poultry by way of stimulating digestive enzymes, exerting antimicrobial, antioxidant, and immune modulation effects, and improving intestinal health and nutrient digestibility [13,14,15].

Rosemary essential oil (REO) is derived from rosemary plant (*Rosmarinus officinalis* L.) belonging to the Lamiaceae family [16]. REO contains major bioactive compounds such as α-pinene, eucalyptol and camphor [17,18]. Studies have shown that adding EOs to the diet of broiler chicken can improve their performance [19,20]. However, incorporating EOs directly into animal diets in free form has limitations as these bioactive compounds are easily degraded and have an unpleasant smell and taste [21]. To address this issue, nanoencapsulation of EOs has been developed as a viable option. In recent years, nanoencapsulation has emerged as a viable technology to improve the efficacy of EOs by increasing their bioavailability, utilization efficiency, masking unpleasant smell and taste, controlled release and enabling precise targeting of these compounds [22]. In our study, we used chitosan biopolymer as nanoencapsulating material because of its natural abundance, biocompatibility, safety, biodegradability, cationic charge, and antimicrobial potential [23]. Additionally, the positive charge of chitosan facilitates increased adhesion to negatively charged microbial membranes, which may increase the nanoencapsulated EO’s antibacterial potency against harmful bacteria.

The present study was undertaken with the explicit aim of evaluating the effect of nanoencapsulated REO on growth performance, nutrient digestibility, carcass traits, meat quality and gene expression of broiler chicken. Growth performance and nutrient digestibility are important indicators of determining the potential of feed additives, whereas evaluation of carcass traits and meat quality are equally important from consumer’s point of view. Moreover, analyzing the changes in gene expression brought about by supplementing nanoencapsulated REO can provide a scientific basis for its use as a feed additive. This study thus aims to contribute to the development of a novel and safe alternative antibiotic feed additive with potential implications for broiler productivity and welfare.

## 2. Materials and Methods

### 2.1. Extraction of REO

The REO was extracted from the aerial parts of rosemary plant (*Rosmarinus officinalis*) at a field station of Council of Scientific and Industrial Research (CSIR), Bonera, Pulwama, India. A mixture of 100 g of rosemary and 1.2 L of distilled water was prepared. According to the Pharmacopoeia, the mixture was subjected to hydro-distillation for 2 h (or until no more EO was recovered) using a Clevenger-type apparatus [24]. The EO was collected, weighed, and then dried using anhydrous sodium sulfate before being kept in sealed vials at 4 °C in the dark until it was needed.

### 2.2. Analysis of REO by Gas Chromatography-Mass Spectrometry

The analysis of REO for the presence of various bioactive compounds was performed by a gas chromatography-mass spectrometry (GC-MS) system (GC-MS/MS-7000D, Agilent Technologies, Palo Alto, CA, USA) equipped with a splitless injector, maintained at a temperature of 280 °C and a detector maintained at the same temperature. An injection volume of 1 μL was used. A HP-5MS UI column (Agilent J&W, Santa Clara, CA, USA) of 30 m length, 0.25 mm internal diameter and 0.25 μm film thickness was used with helium as a carrier gas at a constant flow rate of 1 mL/min. The oven temperature was programmed from 60 °C to 310 °C and 40.5 min was the total run time. The identification of REO bioactive compounds was performed by matching the relative retention indices and mass spectra of those compounds with MS Library (Mass Spectral Library, NIST-17, Gaithersburg, MD, USA).

### 2.3. Nanoencapsulation of REO

**Figure 1:**
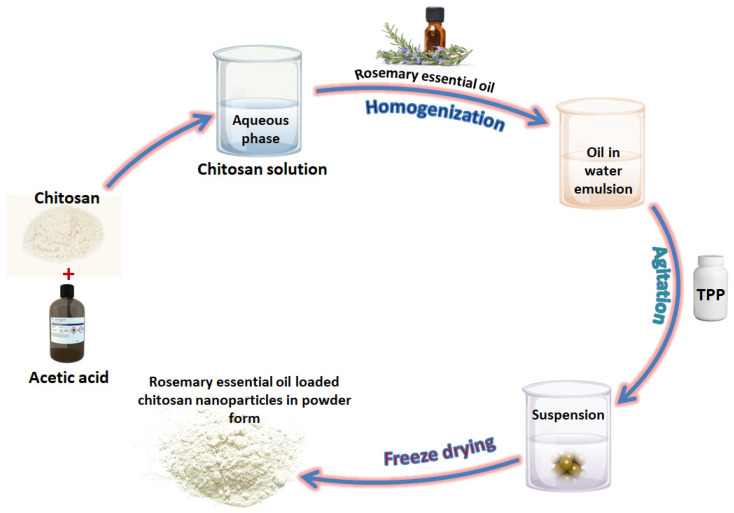
Illustration of nanoencapsulation of rosemary essential oil in chitosan through the ionic gelation method.

Chitosan (purchased from HiMedia laboratories) was used as a nanoencapsulating material. Nanoencapsulation of REO was achieved by the ionic gelation method [25] and illustrated in Figure 1. Chitosan (1 mg/mL) was dissolved in 1% acetic acid (*w*/*v*) and resultant solution was sonicated. Thereafter, it was subjected to centrifugation (Eppendorff-5801R) at 10,000 rpm for 30 min. The supernatant was removed and precipitate filtered through filter paper (1 µm pore size) to obtain a clear aqueous chitosan solution. The obtained solution was then homogenized (WiseTis^®^, Wertheim, Germany) to obtain an emulsion. Sodium triphosphate pentabasic (TTP) solution (Sigma Aldrich, Bangalore, India; 1 mg/mL) was added into the agitated emulsion under constant stirring for 40 min to obtain REO-loaded chitosan nanoparticles. Centrifugation was performed again at 4 °C for 30 min at 10,000 rpm and several washings were given. The ultrasonication was performed thereafter at 40 KHz for 5 min using an ultrasonic probe. Unloaded chitosan nanoparticles were prepared by the same procedure without the addition of REO. The solutions were frozen to −18 °C and subjected to freeze drying (Lyovapour^TM^ L-200, BUCHI, Flawil, Switzerland). The freeze-dried materials were kept in air-tight containers and stored at −18 °C until use.

### 2.4. Scanning Electron Microscopy

The morphological properties of REO-loaded nanoparticles were evaluated through scanning electron microscopy (SEM) (ZEISS Gemini SEM 500, 8203017193, Gaithersburg, UK). The samples were fixed on aluminum stubs using adhesive carbon tape, mounted on the grid and coated with a thin film of gold (sputter coater, Model SC7620) before taking SEM images at 100 kX magnification.

### 2.5. Experimental Design and Diets

The experiment was conducted in accordance with the guidelines of the committee for the purpose of control and supervision of experiments on animals (CPCSEA, New Delhi, India), Government of India and approved by the Institutional Ethics committee of the Faculty of Veterinary Sciences, Shuhama, SKUAST-Kashmir. The 420-day-old unsexed commercial broiler chicks were obtained from a commercial hatchery and brooded together for a week. At one week of age, after weighing, birds were randomly distributed to seven treatments of four replicates (floor pens) each with 15 chicks/replicate. Sawdust was used as litter material and the trial lasted until six weeks of age. The initial temperature of the house was maintained at 95 °F and, thereafter, decreased by 5 °F each week, so as to provide thermo-comfort to the birds. A 24 h light was offered for the initial 3 days followed by a 1 h reduction each day until this decreased to 18 h and was kept constant thereafter. Diets based on corn-soybean were prepared to meet the requirements of the Bureau of Indian Standards (BIS) [26]. The composition of ingredients and nutrients of the basal diet is shown in Table 1. The birds were offered feed and water ad libitum. The dietary treatments comprised control (CON) fed a basal diet only, AB (basal diet + 100 mg enramycin/kg), CS (basal diet + 150 mg chitosan nanoparticles/kg), REO_F100_ and REO_F200_ (basal diet + 100 mg and 200 mg free REO/kg, respectively), and REO_N100_ and REO_N200_ (basal diet + 100 mg/kg and 200 mg nanoencapsulated REO/kg, respectively). For each treatment, feed was prepared on a weekly basis and stored in airtight containers.

### 2.6. Growth Performance and Nutrient Digestibility

The body weight of individual birds and feed intake (FI) per replicate were assessed on a weekly basis and body weight gain (BWG) and the feed conversion ratio (FCR) were calculated accordingly. Replicate-wise mortality was calculated on a daily basis and adjusted for the calculation of BWG, FI and FCR. The metabolism trial lasting for four days was conducted at 35 d of age in metabolic cages utilizing randomly selected eight birds from each treatment (two birds per replicate). During the metabolic trial, net feed consumed by the bird in the respective dietary treatments was recorded and the droppings were collected quantitatively in pre-weighed aluminum dishes and placed into the forced draft hot air oven at 60 ± 5 °C during all the 4 days of collection. The samples of excreta and diets were powdered and stored for further analysis in air tight containers. Dry matter was determined by a standard procedure [27] (method 930.15), crude protein by Kjeldahl acid digestion [27] (method 2001.11), ether extract by Soxhlet [27] (method 991.36), crude fiber by a standard procedure [27] (method 978.10), phosphorous by a standard procedure [27] (method 968.08D) and calcium by Talpatra et al. [28]. The digestibility (%) of these different nutrients was calculated as: (nutrient intake − nutrient excreted)/nutrient intake.

### 2.7. Carcass Characteristics and Physical Meat Quality Traits

At 42 d of age, 2 birds (1 male and 1 female) per replicate were fasted overnight, weighed and slaughtered using the Halal procedure. The dressing percentage, weight of breasts, thighs and abdominal fat percentage was recorded.

The meat quality of breast samples was determined by measuring the pH values [29] of the samples at 0 and 24 h after they were slaughtered. To do so, 10 g portions of the samples were mixed with distilled water and homogenized for 1 min using a homogenizer. The pH values were measured using a digital pH meter (Tanco, India). Additionally, the water holding capacity (WHC) of the minced meat sample was estimated by mixing it with 0.6 M NaCl, holding it at 4 °C for 15 min, and measuring the supernatant fluid after centrifugation at 5000 rpm for 15 min [30]. The weight of frozen meat samples was recorded and noted as the initial weight (W1). The samples were then placed in labelled zip-lock bags and hung at 4 °C for 24 h. After 24 h, the meat samples were weighed again, and the final weight (W2) was recorded.

(1) $$\mathrm{Drip}\;\mathrm{loss}\;(\mathrm{DL})\;\mathrm{was}\;\mathrm{calculated}\;\mathrm{as}:\;\mathrm{Drip}\;\mathrm{loss}\;(\%)=\frac{\left(W1\;-\;W2\right)}{W1}\;\times \;100$$

For 2 min, blending of 15 g of meat samples with 60 mL of phosphate buffer solution (0.05 M; pH 5.8) was performed. The homogenate was then filtered using Whatman filter paper No. 1 for a predetermined duration of 15 min, resulting in a filtrate that was assessed as the extract reserve volume (ERV) [31]. Additionally, the meat samples’ cholesterol level was ascertained, as explained by Wybenga and Pileggi [32]. After extracting 1 g of meat sample in 15 mL of a 2:1 chloroform–methanol combination, the amount of cholesterol in the extract was measured using a spectrophotometer (HITACHI, Tokyo, Japan, UV-Spectrophotometer U-1800) set to 560 nm in wavelength. Using a colorimeter (YS3060), the color coordinates (lightness-L*, redness-a*, and yellowness-b*) of the cross-sectional sections of breast samples were ascertained. Three color readings were taken directly on the surface of muscle from different locations on each sample and then averaged.

### 2.8. Meat Lipid Peroxidation

The lipid peroxidation of meat samples was evaluated through the determination of thiobarbituric acid-reactive substances (TBARS), free fatty acid (FFA) and peroxide values at 0 and 5 d of storage. The approach of Witte et al. [33] with minor changes was used to estimate the TBARS value. Briefly, 10 g of material was triturated for 2 min in a 2 M orthophosphoric acid solution with 25 mL of pre-cooled 20% trichloroacetic acid (TCA). After rinsing with 25 mL cooled distilled water, the contents were quantitatively transferred into a beaker. The contents were filtered via ash-free filter paper after adequate mixing (Whatman filter paper No. 1 supplied by GE Healthcare, Tokyo, Japan). In test tubes, 3 mL of TCA extract (filtrate) was combined with 3 mL of TBA reagent (0.005 M) and left in the dark for 16 h. A volume of 3 mL of 10% TCA and 3 mL of 0.005 M TBA reagent were combined to make a blank sample. Using a UV-VIS spectrophotometer, the absorbance (O.D.) was measured at a fixed wavelength of 532 nm (HITACHI, UV-Spectrophotometer U-1800). By multiplying the O.D. value with the k factor 5.2, the TBARS value was determined as mg malondialdehyde per kg of sample.

Moreover, a small amount of meat (5 g) was mixed with chloroform (30 mL) along with anhydrous sodium sulphate powder. The mixture was then filtered through a No. 1 Whatman paper 40 [34]. For FFA measurement, 0.2% phenolphthalein indicator was added to the chloroform extract followed by titration with 0.1 N alcoholic potassium hydroxide. For PV measurement, glacial acetic acid (30 mL) and potassium iodide solution (2 mL) were added to the chloroform extract (25 mL), and the mixture was allowed to stand for 2 min before adding distilled water and starch solution (1%). Finally, titration was performed with 0.1 N sodium thiosulphate until the end point was reached. The calculations were made as follows:

$$\mathrm{Free}\;\mathrm{fatty}\;\mathrm{acid}\;(\%)=\frac{\;\left(0.1\times \;\mathrm{vol}.\;\mathrm{of}\;\mathrm{KOH}\;\mathrm{consumed}\times 0.282\right)}{\mathrm{sample}\;\mathrm{weight}}\;\times \;100$$

$$\mathrm{Peroxide}\;\mathrm{value}\;(\mathrm{meq}/\mathrm{kg})=\frac{0.1\times \;\mathrm{vol}.\;\mathrm{of}\;\mathrm{sodium}\;\mathrm{thiosulphate}\;\mathrm{consumed}}{\mathrm{sample}\;\mathrm{weight}}\;\times \;100$$

### 2.9. The Gene Expression Study

Samples of jejunum were collected from the birds that had been slaughtered to evaluate the expression of growth genes (mucin-2 and pepT1) and immune genes (TNF-α and IL-10). To quantify mRNA, the jejunal sections were homogenized and RNA was isolated using the Trizol TM method (Invitrogen, Waltham, MA, USA). The purity and concentration of total RNA were then verified using a UV–visible spectrophotometer (at 260 and 280 nm) and 1% agarose gel. A DNase1 Kit was used to rule out genomic DNA contamination, and cDNA was synthesized using the Thermo Scientific Revert Aid first Strand cDNA Synthesis KitTM (Lithuania). The mRNA expression levels of the target genes were determined by real-time PCR (Roche^TM^, Mannheim, Germany) using SYBR green as a fluorescent dye. The primer sequences used are given in Table 2 [35,36,37]. The expression level of the target genes was normalized with respect to a known housekeeping gene, GAPDH, and the results were expressed as fold changes compared to the control groups following the 2^−ΔΔCT^ method [38]. ΔΔCT corresponded to the difference between CT measured for the mRNA level of each tissue and the CT measured for the mRNA level of the reference gene.

ΔΔCT = CT (target gene) − CT (GAPDH).

### 2.10. Statistical Analysis

The data collected were subjected to analysis of variance using one-way ANOVA (SPSS version, 20.0) and the values were expressed as the means and the pooled standard error. The statistical model is given as:

Y_ij_ = µ + T_i_ + e_ij_

where Y_ij_ represents the observation for the dependent variables at the jth replicate in the ith treatment (i = 1 to 7), µ is the overall mean, T_i_ is the fixed effect of treatments (i = 1 to 7), and e_ij_ is the random error.

Duncan’s multiple range test [39] was used to test the significance of difference between means by considering the differences significant at *p* < 0.05.

## 3. Results

### 3.1. Result of Gas Chromatography-Mass Spectrometry

The results of gas chromatography of REO have been presented in Table 3. Results showed the presence of the main components in REO as camphor (28.19%), α-pinene (15.37%) and 1,8-cineole (14.72%), camphene (10.53%), β-pinene (6.04%), bornyl acetate (4.60%) β-phellandrene (4.36%), borneol (3.81%), myrcene (3.55%), and E-β-caryophyllene (2.39%).

### 3.2. Result of Scanning Electron Microscopy

**Figure 2:**
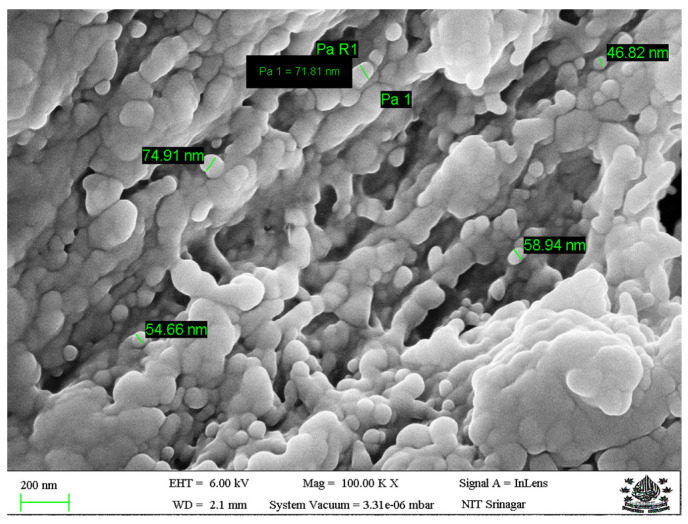
Scanning electron microscopy of rosemary essential oil-loaded chitosan nanoparticles.

The scanning electron microscopy result regarding the morphology of REO-loaded nanoparticles is shown in Figure 2. The REO-loaded nanoparticles were mostly of spherical shape with a smooth surface, with agglomeration at some places. Further, the majority of the particles were less than 100 nm in size.

### 3.3. Effect on Growth Performance

The effects of dietary treatments on growth performance are shown in Table 4. BWG of broiler chicken showed no significance (*p* > 0.05) between dietary treatments and CON from 7 to 21 d of age. There was a significant effect of treatments on BWG when compared with the CON group from 22 to 42 d of age (*p* = 0.035). The highest improvement of 8.39% in the BWG was recorded in the REO_N200_ group than with CON (1381 vs. 1265 g/bird). Overall (7–42 d), a significantly (*p* < 0.01) lower BWG was noticed in the CON group (1742 g/bird) and tended to improve in all other treatments. The CS, REO_F100_ and REO_F200_ groups showed statistically similar values, but lower than the AB and REO_N100_ groups. REO_N200_ had highest BWG (1899 g/bird) than CON and all other dietary treatments.

No significant (*p* > 0.05) effect on FI was observed during different phases and the overall rearing period. From 7 to 21 d of age, no significant (*p* > 0.05) effect on FCR was found among treatments and CON. Afterwards, FCR decreased (*p* < 0.05) in dietary treatments when compared with the CON (22–42 d). During the overall period (7–42 d), FCR reduced significantly (*p* = 0.001), with a peak reduction of 8.47% in the REO_N200_ group (1.62 vs. 1.77), followed by a 6.21% decrease in the AB and REO_N100_ groups (1.66 vs. 1.77) when compared with the CON.

### 3.4. Effect on Nutrient Digestibility

The effects of dietary treatments on nutrient digestibility are given in Table 5. REO supplementation increased (*p* < 0.05) the digestibility of dry matter (DM) and crude protein (CP) at 35 d in broiler chicken when compared with the CON. REO_N100_ and REO_N200_ showed greater improvement than all other groups. Ether extract, crude fiber, calcium, and phosphorus digestibility values showed no difference (*p* > 0.05) among all the dietary treatments and CON.

### 3.5. Effect on Carcass Attributes

The effects of dietary treatments on carcass traits are presented in Table 6. The dressing %, weight of breast and thigh significantly increased (*p* < 0.05) in the REO_N200_ group when compared to other treatments and CON. Moreover, REO_F200_, REO_N100_ and REO_N200_ had a significantly decreased (*p* = 0.001) abdominal fat % than other groups.

### 3.6. Effect on Physical Meat Quality Attributes

The effects of dietary treatments on physical traits of breast meat are illustrated in Table 7. The pH of meat samples at 0 and 24 h after slaughter showed statistically similar values between treatments and CON. WHC and ERV of breast (WHC: *p* = 0.04; ERV: *p* < 0.001) meat tended to increase significantly in the birds of REO_N200_ when compared to CON. DL decreased in breast (*p* = 0.001) samples of the REO_N100_ and REO_N200_ groups when compared to other treatments and CON. REO_N100_ and REO_N200_ supplementation resulted in a significantly lower cholesterol content in meat samples (*p* < 0.001) than in the CON group. No significant difference (*p* > 0.05) was observed in the breast meat color between the dietary treatments and CON.

### 3.7. Effect on Meat Lipid Peroxidation Parameters

The effects of dietary treatments on lipid peroxidation parameters of breast meat are illustrated in Table 8. TBARS, FFA and PV changed by inclusion of various diets and decreased significantly at 0 days of storage (TBARS: *p* = 0.012; FFA: *p* < 0.001; PV: *p* < 0.001) in meat samples of birds belonging to the REO_N100_ and REO_N200_ groups when compared to the CON. At 5 days of storage, the REO_F200_, REO_N100_ and REO_N200_ groups showed reduced TBARS, FFA and PV in breast samples (TBARS: *p* < 0.001; FFA: *p* = 0.035; PV: *p* = 0.029) when compared to other dietary treatments and CON.

### 3.8. Effect on Gene Expression

**Figure 3:**
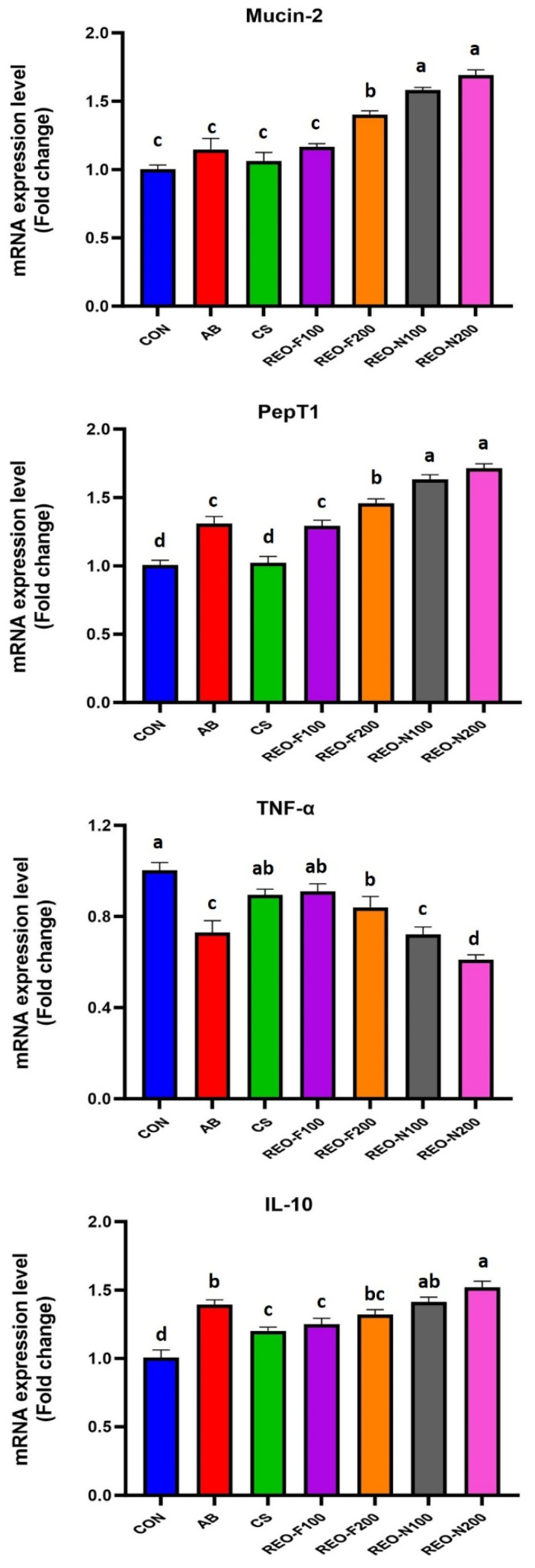
Effect of free and nanoencapsulated rosemary essential oil on gene expression of growth and immune-related genes of broiler chicken. Bars with different upper scripts (a, b, c and d) differ significantly from each other.

The effects of dietary treatments on the gene expression are shown in Figure 3. The expression of the *Mucin 2* gene was upregulated (*p* < 0.001) in REO_F200_-, REO_N100_- and REO_N200_-supplemented groups. The greatest expression was noticed in the REO_N200_ group. *PepT1* gene expression was upregulated (*p* < 0.001) in the REO_N100_ and REO_N200_ groups when compared with the CON, followed by the AB, REO_F100_ and REO_F200_ groups. TNF-α gene expression decreased significantly (*p* < 0.001) in the REO_F200_, REO_N100_ and REO_N200_ groups when compared to the control. The gene expression of IL-10 was higher significantly (*p* < 0.001) higher in all dietary treatments than CON, with maximum upregulation in the REO_N200_ group.

## 4. Discussion

During the starter phase (7–21 d), there was no dietary effect on BWG of birds when compared with the CN. In the finisher phase (22–42 d), dietary treatments showed a significant effect with a 8.39% increment in BWG over CN. For the overall period (7–42 d), BWG tended to augment significantly in AB and nanoencapsulated groups (REO_N100_ and REO_N200_), with a peak increase of 8.11% in REO_N200_ over CN. During the starter phase, in contrast to our results, an improvement in BWG by 5.81% [19] and 5.66% [40] was reported by adding 100 and 200 mg REO in the diet, respectively. In the finisher phase, similar results were observed in previous study [40], who reported 2.55% increase in BWG of broilers. For the overall period, the results found in the present study are in line with other researchers who reported an increase of 4.43% in BWG of broilers by free REO supplementation [19]. Ertas et al. [41] observed an increased BWG in birds fed 200 ppm EOs in the diet compared to control. Ghozlan et al. [42], however, documented that BWG decreased by inclusion of rosemary leaves at 1 and 1.5% in the diet. Other authors also reported that supplementation of free REO at 200 mg/kg diet significantly decreased BWG in broiler chicken [43]. This variation in the results could be due to difference in genotype of plants, soil conditions, time of harvest, maturity of plant and method used for extraction of EOs [44]. The positive effect of REO in BWG could be attributed to the fact that EOs possesses antioxidant and immune modulatory effects [45,46], improve intestinal morphometry [47], stimulate digestive enzyme secretion [48], maintain proper microbiota balance in the gut [49], improve nutrient digestibility [13] and reduce incidence of intestinal diseases [50]. Many authors have attributed the beneficial effect of REO to its major three bioactive compounds which include 1,8-cineole, α-pinene and camphor [22]. In the current study, these positive effects, were augmented considerably by nanoencapsulation process, which increases their solubility and stability and releases EOs in a controlled manner [22].

During all the stages, no effect on FI was noticed between dietary treatments and CON, thus confirming the results of an earlier study [19]. There was no statistical difference in the FI by adding 10 g/kg ground rosemary to layer chicken [51]. These results are in disagreement with other authors [43], who documented that REO inclusion in the diet of broiler chicken at 100 and 200 mg resulted in an increase in FI when compared with the control. FI was reported to improve by 5.13% and 7.84% in 100 and 200 mg/kg diet REO groups, respectively, compared against control [40].

FCR did not differ among dietary treatments and CON during the starter phase (7–21 d). However, during the finisher phase (22–42 d) and the overall period (7–42 d), FCR tended to get better by the influence of dietary treatments when compared with the CON, thus supporting the findings of [19]. These results, however, contradict the observations of Abd El-Latif et al. [43], who supplemented the broiler chicken with 100 and 200 mg REO and noticed increased FCR in these groups when compared with the control. In our study, the best FCR was observed in REO_N200_, which could be attributed to the beneficial effects of nanoencapsulation process.

Nutrient digestibility was found to be influenced by dietary treatments. DM and CP digestibility improved in the birds fed REO in diet when compared with the CON, with the highest values in nanoencapsulated groups. A significant enhancement in DM and CP digestibility in broilers supplemented with garlic or lemon EO at 200 mg/kg diet has been reported [52]. Improved digestibility of nutrients by supplementation of phytogenics has been documented by other authors as well [53]. There was greater nutrient digestibility and retention in broilers fed EOs in the diet [14]. The positive effect on nutrient digestibility has been attributed to the fact that EOs stimulate the digestive secretions of bile, mucus, etc., and improve enzyme activity like trypsin and amylase [54,55]. Moreover, EOs limit the adherence of pathogens to intestinal wall, improve intestinal morphology, balance gut flora, and exert antioxidant and immunomodulatory effects [56,57]. Increased nutrient digestibility results in better feed efficiency, thus supporting the findings of this study recorded in REO groups, especially REO_N200_.

There was a significant improvement in the dressed % of broilers in the groups supplemented with nanoencapsulated REO (100 and 200 mg/kg) in the diet when compared with the control group. These results are in agreement with Attai et al. [58], who reported a significant increase in the dressing % of birds fed encapsulated commercial EO in the diet at 50, 100 and 150 mg/kg feed. Improvement in the dressing percentage by supplementation of EOs was also reported earlier [59]. In the present study, REO in the free form had no significant effect on the dressed weight of broiler chicken compared to the control group. No significant change in the dressed yield between control and REO- supplemented groups at 100 or 200 mg/kg feed has been documented [40]. In the present study, the breast and thigh weight improved significantly particularly in nanoencapsulated REO groups when compared to the control. Gharejanloo et al. [40] also reported that supplementation of turmeric EO at 75 and 150 mg/kg diet significantly improved the relative weight of breasts and thighs in broiler chicken compared to those of control birds. These authors attributed the improvement in the weight of valuable muscles to the possible effects of EOs on the production of musculature in broiler chicken. In this study, the abdominal fat % decreased significantly in nanoencapsulated REO groups compared to the control. Earlier authors [60] also reported that the abdominal fat percentage was decreased by feeding REO at 300 mg/kg diet of broilers.

The inclusion of REO (free and nanoencapsulated) had no effect on the pH level of breast meat. Other authors [61] observed that supplementation of quail rations with 100 mg/kg thyme extract had no effect on the pH value in comparison to the control birds. The pH level of good-quality broiler meat has been reported to range from 5.9 to 6.2 [62]. One of the critical traits used by meat industry for assessment of meat quality is its pH value [63].

In the present study, a significant increase in the WHC and ERV and a significant decrease in the DL in the birds fed nanoencapsulated REO in the diet were noted. Popović et al. [64] supplemented broiler birds a mixture of EOs and reported a significant increase in WHC when compared with the control. In contrast, no significant differences in pH, WHC and DL of thigh meat were recorded in birds fed diets supplemented with lavender extract at 200, 300 and 400 mg/kg [65]. Likewise, feeding fennel EO at 200 mg/kg diet to the birds resulted in no significant effect on breast and thigh meat pH and DL compared to the control [66].

The cholesterol content in breast meat significantly decreased in birds fed REO in the diet, with the highest reduction in the nanoencapsulated groups. These results are in accordance with others [67], who reported that use of phytogenics in feed resulted in a reduction in cholesterol content in broiler chicken meat. The cholesterol-lowering effect of EOs in broiler chicken meat has been attributed to the presence of various bioactive compounds which decreases HMG-CoA reductase protein expression, leading to reduction in the serum cholesterol levels [68]. Others relate it to the effect of EOs in decreasing the activity of various enzymes involved in cholesterol production [69]. In contrast, there was no significant effect on the cholesterol concentration of breast meat following supplementation of fennel EO in broiler chicken diet [66].

The coloration of fresh chicken meat is considered a significant factor in consumer preference for meat [70]. REO inclusion in the diet, either in free or nanoencapsulated form, were observed to maintain color coordinates of breast meat in broiler chicken. Gumus et al. [71], fed broiler chicken with thyme EO at 150 and 300 mg/kg and REO at 100 and 200 mg/kg diet and documented that these EOs did not show any adverse effect on the color parameters of breast and drumstick meat. Others [72] also reported that various EOs did not alter color coordinates of broiler chicken meat.

The degree of meat lipid peroxidation was determined by estimating the meat TBARS value, FFA and PV. Poultry meat is rich in highly unsaturated fatty acids so it has a higher rate of oxidative deterioration than other types of meat. The TBARS method is used to define the scale of rancidity, souring that occurs as a result of antioxidation in fat and fatty parts of meat and determined on the basis of formation of products like MDA from the oxidative damage [73]. The FFA value is the measure of hydrolytic rancidity due to lipidic enzyme activity of microbial and muscle origin resulting in accumulation of FFA, leading to undesirable effects in foods [74]. Peroxide values involve measurement of peroxides and hydroperoxides during the initial stages of lipid oxidation [32]. The results in the present study suggested that there was a significant decrease in TBARS, FFA and PV in the meat of birds supplemented with nonencapsulated REO in the diet. Further, this decrease in the values of TBARS, FFA and peroxides was found to be dose dependent. Shaltout et al. [75] also reported a dose-dependent decrease in the TBARS value by inclusion of thyme EO in the diet of broiler chicken. Supplementation of REO at 300 mg/kg diet significantly decreased the TBARS value in thigh muscle at 9 days after storage [60]. Hamada et al. [76] reported that the FFA value decreased in breast meat of broiler chicken fed thyme EO in the diet when compared with the control. Adding dietary thyme EO decreased the peroxide value in the meat of broiler chicken than the control group [58]. Supplementation of broiler chicken rations with 120 ppm of REO and 500 ppm of thyme EO reduced lipid oxidation in the meat [77]. The antioxidant properties could be attributed to the presence of various bioactive compounds in EOs possessing radical scavenging properties. Further, EOs have the ability to inactivate free radicals produced during the auto oxidation process [78]. Phytogenic products exert defense against lipid peroxidation by influencing the activity of antioxidant enzymes which can decrease the inflammatory agents such as ROS reactive oxygen species, protecting cells and tissues from oxidation [79]. EOs reduce lipid oxidation and extend shelf life of broiler meat during storage [80].

The influence of REO supplementation on the expression of growth-related genes (*Mucin-2* and *PepT1*) was evaluated. *Mucin-2* gene expression was upregulated in all the groups fed REO in the diet when compared with the CON with peak elevation in nanoencapsulated groups. These results corroborate the findings of others, who reported increased expression of *Mucin-2* gene by the influence of free and encapsulated dietary EOs [81,82,83]. *Mucin-2* gene encodes for the secretory mucin, which forms the major component of mucus layer covering intestinal epithelium [84]. The mucus layer acts as first line of defense against harmful antigens and invading pathogens [85]. In addition to acting as physical barrier, mucus facilitates formation of SIgA-mediated immune defense. This not only hampers invasion of intestinal epithelial cells by gut flora but also selectively enhance adherent growth of beneficial flora [86]. Therefore, the elevated expression of *Mucin-2* gene might have resulted in the strong intestinal defense against pathogens and subsequent increment in the performance of birds as recorded in the present study in REO groups. Further, we observed that the expression of *PepT1* gene enhanced in REO-supplemented groups, more so in nanoencapsulated ones. He et al. [87] also documented upregulation of PepT1 under the dietary influence of EOs in broilers. *PepT1* gene is involved in transport of peptides in the intestines [88]. Upregulation in the *PepT1* gene expression increases the influx of peptides into the intestinal epithelial cells [89]. These findings partly support the superior growth performance of nanoencapsulated REO-supplemented groups in this study.

The effect of REO on the expression of immune-related genes (TNF-α and IL-10) was also studied and it was observed that REO, especially in nanoencapsulated form, significantly downregulated the mRNA expression of TNF-α and upregulated the mRNA expression of IL-10 when compared with the control. Similar results were reported with the supplementation of nanoemulsion of eugenol EO by previous authors [90]. TNF-α is a proinflammatory and IL-10 is a anti-inflammatory cytokine. During bacterial invasion in the intestinal epithelial cells, gastrointestinal immune cells secrete cytokines, which play potential roles in the immune response against pathogens [91]. TNF-α regulates host immune response against many pathogens through differentiation and proliferation of immune cells by recruiting antimicrobial cells like neutrophils and macrophages [92]. Decreased expressions of TNF-α noticed in the present study indicated that the antibacterial effect of REO might have decreased the pathogenic load and subsequent inflammatory reaction in the intestinal tract of birds. IL-10 has the opposite effect and plays a vital role in suppressing the inflammatory and immune responses [93]. Therefore, the upregulation in the mRNA expression of IL-10 gene in REO groups suggested the suppression of excessive inflammation and maintenance of intestinal immune homeostasis, thereby indicating the strong anti-inflammatory properties of REO, especially in nanoencapsulated form.

## 5. Conclusions

In this study, adding 200 mg/kg nanoencapsulated rosemary essential oil (REO) to broiler diets improved body weight gain and feed efficiency without affecting feed consumption. The groups that received nanoencapsulated REO had enhanced nutrient digestibility, better carcass traits, and superior meat quality. In addition, increased expression of growth and anti-inflammatory genes (mucin-2, Pep-T1, IL-10) and decreased expression of pro-inflammatory genes (TNF-α) were recorded in nanoencapsulated groups. The findings suggest that nanoencapsulation as a novel tool is required to maximize the benefits from bioactive compounds of rosemary essential oil. In conclusion, nanoencapsulated REO, especially at 200 mg/kg feed, could be an effective alternative to conventional antibiotic growth promoters in broiler chicken.

## Figures and Tables

**Table 1:** Ingredients and nutrient composition of broiler chicken diets.

Ingredients (g/kg)	Starter (7–21 Day)	Finisher (22–42 Day)
Corn	550	586
Soybean Meal	350	320
Fish Meal	40	20
Vegetable Oil	30	40
Limestone	7.00	10.00
Di-Calcium Phosphate	15.00	16.00
Salt	3.00	3.00
DL-Methionine	1.10	1.00
Lysine	1.30	1.40
Trcae Mineral Premix ^1^	1.00	1.00
Vitamin Premix ^2^	1.50	1.50
Nutrient composition		
Crude Protein (%) *	22.3	20.4
Metabolizable Energy (Kcal/kg) **	3062	3150
Crude Fiber (CF) (%) *	4.41	4.86
Ether Extract (%) *	7.36	8.59
Calcium (%) *	1.41	1.23
Available P (%) *	0.70	0.66
Lysine (%) **	1.24	1.09
Methionine (%) **	0.51	0.46

^1^ Trace mineral premix (mg/kg diet): Mg 300, Mn 55, I 0.4, Fe 56, Zn 30 and Cu 4. ^2^ Vitamin premix (per kg diet): Vitamin A 8250 IU, Vitamin D3 1200 ICU, Vitamin K 1 mg, Vitamin E 40 IU, Vitamin B1 2 mg, Vitamin B2 4 mg, Vitamin B1 210 mg, Niacin 60 mg, Pantothenic acid 10 mg, and choline 500 mg. * Analyzed values; ** calculated values.

**Table 2:** List of primers used for quantitative real-time PCR.

Gene	Primer Sequence (5′→3′)	Reference
Mucin-2	CTGGCTCCTTGTGGCTCCTC AGCTGCATGACTGGAGACAACTG	[35]
PepT1	ACGCATACTGTCACCATCAAGAAT TCCAAAAGTCGTGTCACCCATA	[35]
TNF-α	CCTGCTGGGGGAATGCTAGG AGCGTTGTCTGCTCTGTAGC	[36]
IL-10	CAGACCAGCACCAGTCATCAG ATCCCGTTCTCATCCATCTTCTCG	[37]
GAPDH	GTCAGCAATGCATCGTGCA GGCATGGACAGTGGTCATAAGA	[35]

**Table 3:** Percentage of major bioactive compounds in rosemary essential oil *.

Bioactive Compound	Retention Index	Percentage
α-Pinene	929	15.37
Camphene	945	10.53
β-Pinene	978	6.04
Myrcene	991	3.55
β-Phellandrene	1025	4.36
1,8-Cineole	1031	14.72
Camphor	1127	28.19
Borneol	1144	3.81
Bornyl acetate	1262	4.60
E-β-Caryophyllene	1406	2.39

* Identification by gas chromatography-mass spectrometry, National Institute of Standards and Technology (NIST, USA).

**Table 4:** Effect of free and nanoencapsulated rosemary essential oil on the performance of broiler chicken.

Parameter	Dietary Treatments							SEM	*p*-Value
	CON	AB	CS	REO_F100_	REO_F200_	REO_N100_	REO_N200_		
BWG, g									
7–21 d	477	494	478	480	486	497	518	4.78	0.233
22–42 d	1265 ^b^	1356 ^ab^	1298 ^ab^	1301 ^ab^	1321 ^ab^	1351 ^ab^	1381 ^a^	12.01	<0.001
7–42 d	1742 ^c^	1850 ^ab^	1776 ^bc^	1781 ^bc^	1807 ^bc^	1848 ^ab^	1899 ^a^	12.56	0.035
FI, g									
7–21 d	599	587	591	593	593	597	594	2.60	0.942
22–42 d	2490	2482	2485	2474	2478	2462	2474	8.69	0.992
7–42 d	3088	3069	3076	3067	3071	3058	3068	8.26	0.987
FCR									
7–21 d	1.26	1.19	1.24	1.24	1.22	1.20	1.15	0.013	0.343
22–42 d	1.97 ^a^	1.83 ^ab^	1.92 ^ab^	1.91 ^ab^	1.88 ^ab^	1.82 ^ab^	1.80 ^b^	0.017	0.048
7–42 d	1.77 ^a^	1.66 ^bc^	1.73 ^ab^	1.72 ^ab^	1.70 ^ab^	1.66 ^bc^	1.62 ^c^	0.012	0.001

Means with different superscripts in the same row differ significantly (*p* < 0.05); CON (control) fed basal diet only, AB (antibiotic) fed basal diet + 10 g/ton enramycin, CS (Chitosan) fed basal diet + 150 mg/kg chitosan nanoparticles, REO_F100_ and REO_F200_ fed basal diet + 100 mg/kg and 200 mg/kg free REO, respectively, REO_N100_ and REO_N200_ fed basal diet + 100 mg/kg and 200 mg/kg nanoencapsulated REO, respectively; BWG: body weight gain; FI: feed intake; FCR: feed conversion ratio; SEM: standard error of the mean.

**Table 5:** Effect of free and nanoencapsulated rosemary essential oil on nutrient digestibility in broiler chicken.

Parameter (%)	Dietary Treatments							SEM	*p*-Value
	CON	AB	CS	REO_F100_	REO_F200_	REO_N100_	REO_N200_		
Dry matter	70.46 ^c^	73.65 ^b^	70.68 ^c^	71.32 ^b^	72.07 ^b^	74.87 ^ab^	76.37 ^a^	0.49	<0.001
Crude protein	68.97 ^d^	72.30 ^bc^	69.22 ^d^	72.02 ^c^	71.10 ^bc^	73.61 ^ab^	75.46 ^a^	0.55	<0.001
Ether extract	73.49	74.97	73.34	74.30	75.07	75.63	76.44	1.00	0.988
Crude fiber	18.09	17.14	18.45	18.72	18.86	19.02	19.70	0.44	0.868
Calcium	46.42	48.09	46.88	48.28	48.50	49.18	49.68	0.76	0.940
Phosphorous	51.01	52.92	51.58	52.56	52.70	53.30	54.06	0.87	0.984

Means with different superscripts in the same row differ significantly (*p* < 0.05); CON (control) fed basal diet only, AB (antibiotic) fed basal diet + 10 g/ton enramycin, CS (Chitosan) fed basal diet + 150 mg/kg chitosan nanoparticles, REO_F100_ and REO_F200_ fed basal diet + 100 mg/kg and 200 mg/kg free REO, respectively, REO_N100_ and REO_N200_ fed basal diet + 100 mg/kg and 200 mg/kg nanoencapsulated REO, respectively; SEM: standard error of the mean.

**Table 6:** Effect of free and nanoencapsulated rosemary essential oil on carcass traits of broiler chicken.

Parameter	Dietary Treatments							SEM	*p*-Value
	CON	AB	CS	REO_F100_	REO_F200_	REO_N100_	REO_N200_		
PSLW (g)	1912.89 ^c^	1986.45 ^ab^	1928.82 ^bc^	1939.61 ^bc^	1952.70 ^bc^	1994.48 ^ab^	2031.63 ^a^	10.70	0.009
Dressing (%) *	70.36 ^c^	72.48 ^a^	70.41 ^c^	70.74 ^bc^	70.69 ^bc^	72.30 ^ab^	72.49 ^a^	0.279	0.041
Breast (%) *	18.03 ^c^	20.81 ^ab^	18.06 ^c^	18.65 ^bc^	19.21 ^abc^	20.90 ^ab^	21.49 ^a^	0.383	0.020
Thigh (%) *	8.98 ^b^	10.46 ^a^	9.06 ^b^	9.39 ^ab^	9.50 ^ab^	10.28 ^a^	10.49 ^a^	0.177	0.028
Abdominal fat (%) *	1.24 ^a^	1.25 ^a^	1.22 ^ab^	1.21 ^abc^	1.19 ^bc^	1.17 ^cd^	1.14 ^d^	0.009	0.001

Means with different superscripts in the same row differ significantly (*p* < 0.05); CON (control) fed basal diet only, AB (antibiotic) fed basal diet + 10 g/ton enramycin, CS (Chitosan) fed basal diet + 150 mg/kg chitosan nanoparticles, REO_F100_ and REO_F200_ fed basal diet + 100 mg/kg and 200 mg/kg free REO, respectively, REO_N100_ and REO_N200_ fed basal diet + 100 mg/kg and 200 mg/kg nanoencapsulated REO, respectively; PSLW: pre-slaughter live weight; * percent live weight; SEM: standard error of the mean.

**Table 7:** Effect of free and nanoencapsulated rosemary essential oil on physical traits of breast meat of broiler chicken.

Parameter		Dietary Treatments						SEM	*p*-Value
	CON	AB	CS	REO_F100_	REO_F200_	REO_N100_	REO_N200_		
^0^ pH	5.88	5.84	5.85	5.90	5.93	5.98	6.04	0.036	0.949
^24^ pH	5.81	5.78	5.82	5.87	5.89	5.92	5.95	0.039	0.957
WHC (%)	54.17 ^b^	53.96 ^b^	54.03 ^b^	54.52 ^b^	54.75 ^ab^	55.70 ^ab^	56.41 ^a^	0.249	0.04
DL (%)	3.11 ^a^	3.14 ^a^	3.10 ^a^	3.05 ^ab^	3.01 ^ab^	2.92 ^bc^	2.85 ^c^	0.024	0.001
ERV (%)	25.17 ^b^	25.11 ^b^	25.24 ^b^	25.85 ^b^	26.42 ^b^	28.55 ^a^	29.37 ^a^	0.347	<0.001
Cholesterol (mg/kg)	69.82 ^a^	69.57 ^a^	70.01 ^a^	68.77 ^a^	64.92 ^ab^	62.66 ^b^	60.55 ^c^	0.688	<0.001
L*	51.30	52.12	51.63	52.46	51.97	53.12	52.24	0.280	0.770
a*	3.19	3.51	3.77	3.31	3.43	3.24	3.90	0.139	0.836
b*	5.63	5.46	5.39	5.43	5.72	5.52	5.29	0.168	0.998

Means with different superscripts in the same row differ significantly (*p* < 0.05); CON (control) fed basal diet only, AB (antibiotic) fed basal diet + 10 g/ton enramycin, CS (Chitosan) fed basal diet + 150 mg/kg chitosan nanoparticles, REO_F100_ and REO_F200_ fed basal diet + 100 mg/kg and 200 mg/kg free REO, respectively, REO_N100_ and REO_N200_ fed basal diet + 100 mg/kg and 200 mg/kg nanoencapsulated REO, respectively; WHC—water holding capacity, DL—Drip loss, ERV—Extract reserve volume; L*—lightness; a*—redness; b*—yellowness; SEM: standard error of the mean.

**Table 8:** Effect of free and nanoencapsulated rosemary essential oil on lipid peroxidation parameters of breast meat of broiler chicken.

Parameter	Storage Day		Dietary Treatments						SEM	*p*-Value
		CON	AB	CS	REO_F100_	REO_F200_	REO_N100_	REO_N200_		
TBARS (mgMDA/kg)	0	0.73 ^a^	0.71 ^a^	0.72 ^a^	0.68 ^a^	0.65 ^a^	0.53 ^b^	0.45 ^c^	0.023	0.012
	5	1.59 ^a^	1.58 ^a^	1.60 ^a^	1.56 ^ab^	1.50 ^b^	1.42 ^c^	1.35 ^d^	0.021	<0.001
FFA (%)	0	0.24 ^a^	0.22 ^a^	0.23 ^a^	0.20 ^a^	0.19 ^a^	0.13 ^b^	0.08 ^c^	0.013	<0.001
	5	1.37 ^a^	1.39 ^a^	1.36 ^a^	1.34 ^a^	1.28 ^b^	1.19 ^c^	1.12 ^d^	0.022	0.035
PV (mEq/kg)	0	1.03 ^a^	1.05 ^a^	1.04 ^a^	0.99 ^a^	0.97 ^a^	0.82 ^b^	0.76 ^b^	0.025	<0.001
	5	2.84 ^a^	2.82 ^a^	2.85 ^a^	2.78 ^ab^	2.73 ^b^	2.64 ^c^	2.57 ^c^	0.023	0.029

Means with different superscripts in the same row differ significantly (*p* < 0.05); CON (control) fed basal diet only, AB (antibiotic) fed basal diet + 10 g/ton enramycin, CS (Chitosan) fed basal diet + 150 mg/kg chitosan nanoparticles, REO_F100_ and REO_F200_ fed basal diet + 100 mg/kg and 200 mg/kg free REO, respectively, REO_N100_ and REO_N200_ fed basal diet + 100 mg/kg and 200 mg/kg nanoencapsulated REO, respectively; TBARS: thiobarbituric acid-reactive substances; FFA: free fatty acid value; PV: peroxide value; SEM: standard error of the mean.

## Data Availability

The original contributions presented in the study are included in the article, further inquiries can be directed to the corresponding author.
